# Optimizing papillary incision design in molar periodontal regenerative therapy: A case study

**DOI:** 10.1002/cap.70011

**Published:** 2026-04-15

**Authors:** Shunichi Shibazaki, Satoru Morikawa, Yuichiro Ihara, Taneaki Nakagawa

**Affiliations:** ^1^ Department of Dentistry and Oral Surgery Keio University School of Medicine Tokyo Japan

**Keywords:** microsurgery, molar, periodontal diseases, regeneration, surgical flaps, wound healing

## Abstract

**Background:**

While the simplified papilla preservation flap is commonly recommended in the molar region, it may not be optimal when blood flow and microsurgical visibility are considered. This case study aimed to evaluate the relationship between surgical visibility and interdental incision designs in the molar region and to propose a decision‐making framework for interdental papilla incision design based on the extent of flap elevation and blood supply.

**Methods:**

Three cases of periodontal regenerative therapy in the molar regions were performed using different flap designs: extended flap with circumferential defect (3 mm depth) and minimally invasive surgical technique (MIST) and modified MIST (M‐MIST) with combination defects (4 mm depth), both incorporating interdental incision designs with a buccal or palatal shift. Clinical parameters and radiographic outcomes were assessed at baseline and 6 months postoperatively.

**Results:**

The initial probing pocket depths of 10, 9, and 8 mm were reduced to 4, 4, and 3 mm, respectively, with radiographic evidence of bone‐filling in all cases. Cases using MIST and M‐MIST demonstrated superior primary healing compared with the extended flap case. Interdental incision designs with a buccal or palatal shift improved visibility and operability during microsurgery in molar regions.

**Conclusions:**

This case study showed that buccal‐ or palatal‐shifted interdental incision designs were effective for molar periodontal regenerative therapy under operating microscopes. The decision‐making process for interdental incision designs should consider the inter‐root distance as well as the extent of flap elevation and its impact on blood supply.

**Key points:**

- Buccal‐ or palatal‐shifted interdental incision designs using an appropriate flap design can be successfully applied to molar regions.
- Microscope‐enhanced visibility with buccal‐ or palatal‐shifted interdental incision designs improves surgical precision and accessibility.
- Preservation of the blood supply is crucial for optimal wound healing.

**Plain language summary:**

Successful periodontal regenerative therapy in molar regions requires adequate surgical access and preservation of the interdental papilla. Although various minimally invasive techniques are available, accessing deep bone defects in molar regions while maintaining blood supply to the papilla remains challenging. In this context, interdental papilla incision designs that shift the incision line to the buccal or palatal aspect—such as the papilla preservation technique, modified papilla preservation technique, and triangle papilla access approach—are considered to contribute to improved visibility in periodontal regenerative therapy in the molar region using the operating microscope (OM). Building on this, the present case study proposes a new decision‐making framework for interdental papilla incision design, with an emphasis on enhancing visibility under the OM and considering the anatomical relationship between flap design and blood supply.

## INTRODUCTION

Vertical bone defects are significant risk factors for tooth loss in patients with periodontal disease. Studies have shown that teeth with vertical bone defects exceeding 4 mm have a 70% likelihood of being lost within 10 years.^1^ Additionally, teeth with Class II furcation involvement are approximately six times more likely to be lost than healthy teeth.^2^ Thus, the high rate of tooth loss in teeth with vertical bone defects or furcation involvement underscores the importance of promptly and effectively addressing these defects. The surgical intervention threshold for molars occurs at a notably lower probing pocket depth (PPD) of 4.5 mm, compared with 6.4 mm for anterior teeth and 7.3 mm for premolars, indicating the need for earlier surgical intervention in these areas.^3^ However, the structural complexity of molars, combined with their posterior location and unique anatomical features, makes them particularly challenging for regenerative therapy. These challenges are further complicated by the varied morphological patterns of intrabony defects, including one‐, two‐, and three‐wall and circumferential defects, which significantly impact surgical approach selection and regenerative outcomes.^4^

Successful periodontal regenerative therapy requires the formation of a blood clot and the protection of its surrounding space for its maintenance and stability. Consequently, the blood clot stabilizes on the root surface, facilitating regeneration.^5^ When primary closure is compromised, blood clot detachment from the root surface may occur, potentially leading to epithelial downgrowth and impaired regeneration. Therefore, achieving and maintaining primary closure is a fundamental requirement for successful regenerative therapy.^6^, ^7^

Various papillary incision techniques have been developed to achieve an optimal primary closure of the interdental area.^8^, ^9^, ^10^ The papilla preservation technique (PPT),^8^ modified papilla preservation technique (MPPT),^9^ and simplified papilla preservation flap (SPPF)^10^ have contributed to the evolution of surgical approaches to periodontal regenerative therapy. More recently, the triangle papilla access approach (T‐PAA),^11^ specifically designed for microsurgical procedures, has been developed. The clinical decision between these techniques traditionally depends on the inter‐root distance, with 2 mm serving as the critical threshold.^10^

In addition, minimally invasive surgical technique (MIST)^7^ and modified MIST (M‐MIST),^12^ developed based on the use of the operating microscope (OM), have been shown to facilitate favorable primary closure.

In surgical procedures using the OM, securing a direct view is essential. However, compared with the anterior region, the molar region is more affected by anatomical factors, such as the buccinator muscle and limited mouth opening, thereby increasing the surgical difficulty. Thus, enhancing surgical visibility should be considered an important factor when determining the incision line in microsurgical procedures.

In this case study, interdental incision designs in molar periodontal regenerative therapy under the OM were evaluated from the perspectives of both surgical visibility and blood supply. In addition to the conventional criterion of inter‐root distance, we propose a new decision‐making framework for interdental incision designs that incorporates the concept of enhanced visibility under the OM.

## CASE PRESENTATION

This case study included three patients (two men and one woman). All patients underwent initial therapy consisting of strict oral hygiene instructions, motivation, scaling, and root planing. The full‐mouth plaque score and full‐mouth bleeding score were strictly maintained below 15%.^13^

Written informed consent for surgery and inclusion in this case report were obtained preoperatively. This case study has been reported in accordance with the PROCESS guidelines.^14^ All surgeries were performed by a board‐certified periodontist (Shunichi Shibazaki) using OMs.

### Case 1

**FIGURE 1:**
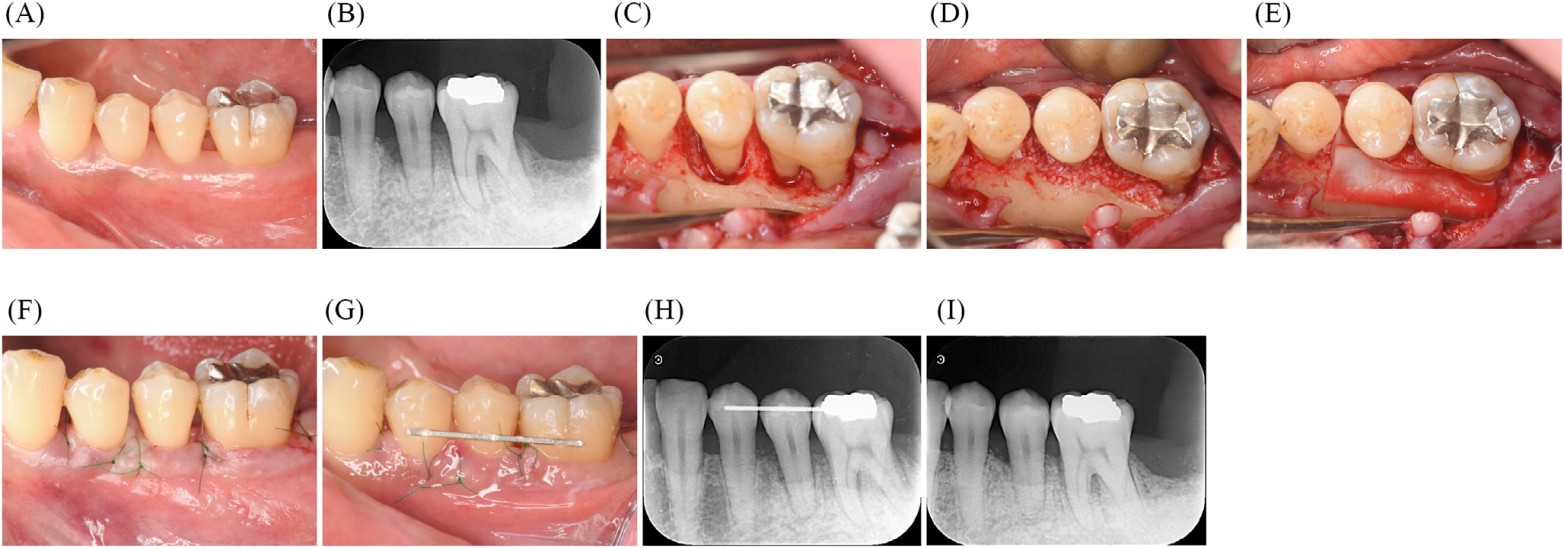
Clinical and radiographic presentation of periodontal regenerative therapy using an extended flap with MPPT in the mandibular molar region. (A) Preoperative clinical presentation of the interdental area between teeth #19 and #20. (B) Preoperative radiograph showing a vertical bone defect at the mesial aspect of tooth #20. (C) Circumferential bone defect pattern. (D) Placement of DBBM in the bone defect. (E) Placement of resorbable membrane (Bio‐Gide). (F) Immediate postoperative buccal view showing primary closure. (G) 1‐week postoperative buccal view showing a slight gingival recession between teeth #19 and #20. (H) Immediately postoperative radiograph. (I) 2‐year follow‐up radiograph showing bone fill. MPPT: modified papilla preservation technique; DBBM: deproteinized bovine bone mineral.

A 58‐year‐old male patient with no history of periodontal treatment presented to Keio University Hospital in January 2022. He had no significant medical conditions that could affect his treatment and no history of smoking. After the initial periodontal therapy and re‐evaluation, a 10 mm PPD was observed, extending from the mesial aspect of tooth #20 to the lingual aspects of teeth #19 and #20 (Figure 1A). Dental radiograph revealed a vertical bone defect in the mesial aspect of tooth #20 (Figure 1B).

Periodontal regenerative therapy was performed on teeth #19 and #20. Incisions were made using a microsurgical blade^*^, extending from the distal aspect of tooth #19 to the mesial line angle of tooth #22. The interdental papilla between teeth #19 and #20 was accessed with MPPT. The full‐thickness flap was elevated to expose the defect‐associated residual bone crest. The morphology of the bone defects in teeth #19 and #20 exhibited a circumferential pattern, extending from the mesial to the lingual side and wrapping around the distal side (Figure 1C). The depth of the bone defect was approximately 3 mm. Calculus was removed using an ultrasonic scaler, followed by root surface debridement using a hand scaler. Emdogain^†^ was delivered, followed by the placement of Bio‐Oss^‡^ to maintain space in this circumferential defect (Figure 1D). The site was covered with a resorbable membrane^§^. The flap was repositioned and sutured using reverse cutting; 3/8 Circle 13 mm 6‐0 nylon sutures^**^ (Figure 1E). Adjacent teeth were splinted immediately postoperatively using wire and resin cement^††^ to stabilize the blood clot. The sutures were removed 7 days postoperatively. Teeth #20 and #21 exhibited good primary healing. However, the area between teeth #19 and #20 showed a slight gingival recession (Figure 1F,G). The Early Wound Healing Index (EHI) score was 4.^15^ At the 2‐year postoperative evaluation, PPD decreased from 10 to 4 mm, and dental radiograph showed evidence of bone fill (Figure 1H,I).

### Case 2

**FIGURE 2:**
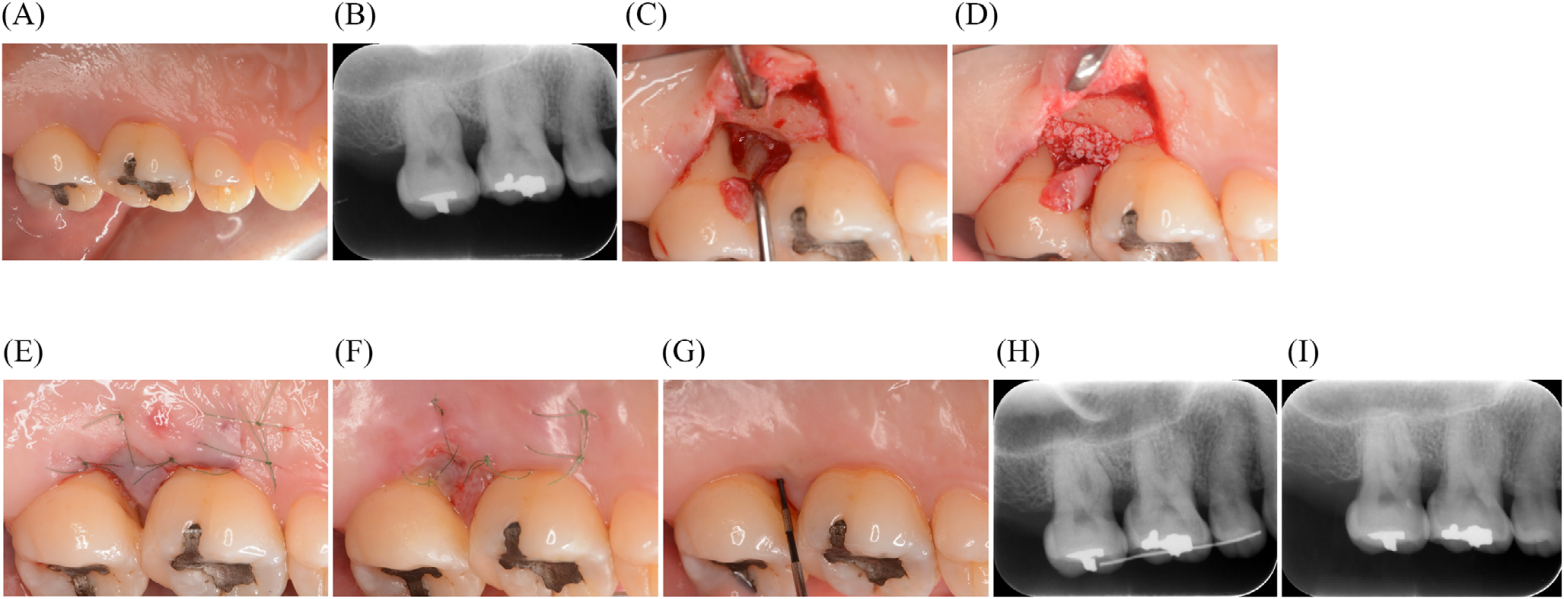
Clinical and radiographic presentation of periodontal regenerative therapy using MIST in the maxillary molar region. (A) Preoperative clinical presentation of tooth #2. (B) Preoperative radiograph showing vertical bone defect at the mesial aspect. (C) 2‐wall bone defect after debridement. (D) Placement of DBBM. (E) Immediate postoperative palatal view showing primary closure. (F) Palatal view showing primary healing at 1‐week postoperative. (G) Clinical presentation at 2‐year follow‐up. (H) Immediate postoperative radiograph. (I) 2‐year follow‐up radiograph showing bone fill. DBBM, deproteinized bovine bone mineral; MIST, minimally invasive surgical technique.

A 45‐year‐old female patient with no history of periodontal treatment presented to Keio University Hospital in August 2022. The patient had no relevant medical or smoking history. After initial periodontal therapy and re‐evaluation, a 9‐mm PPD was observed at the mesiopalatal aspect and 5 mm at the mesiobuccal aspect of tooth #2 (Figure 2A). Dental radiograph revealed a vertical bone defect on the mesial aspect of tooth #2 (Figure 2B).

Given that the bone defect extended beyond the interdental aspect only in the mesial direction of #2, involving both the buccal and palatal regions, periodontal regenerative therapy was performed on tooth #2 using MIST with a papillary triangle and palatal flap. In this case, palatal access was selected to achieve better visualization. Furthermore, compared with buccal access, it allows for less elevation of the interdental papilla, possibly preserving blood supply. The incision on the interdental papilla between teeth #2 and #3 was made using a microsurgical blade. Additionally, to achieve better access, a vertical incision was made on the mesiopalatal aspect of tooth #3 using a surgical blade^‡‡^. The interdental papilla was elevated to reach and expose the residual bone crest of the buccal aspect. The bone defect had a 2‐wall defect configuration (Figure 2C and Videos S1, S2). The depth of the bone defect was approximately 4 mm. After calculus removal and root surface debridement, Emdogain was delivered, followed by Bio‐Oss placement to maintain space in this predominantly 2‐wall defect (Figure 2D, Video S3). Subsequently, the flap was repositioned and sutured using reverse cutting; 3/8 Circle 13 mm 6‐0 nylon sutures (Figure 2E,F and Video S4). Adjacent teeth were splinted immediately postoperatively using wire and resin cement to stabilize the blood clot. The sutures were removed 7 days postoperatively. Good primary healing was achieved. EHI score was 2. At the 2‐year postoperative evaluation, PPD improved from 9 to 4 mm (Figure 2G), and dental radiograph showed evidence of bone fill (Figure 2H,I).

### Case 3

**FIGURE 3:**
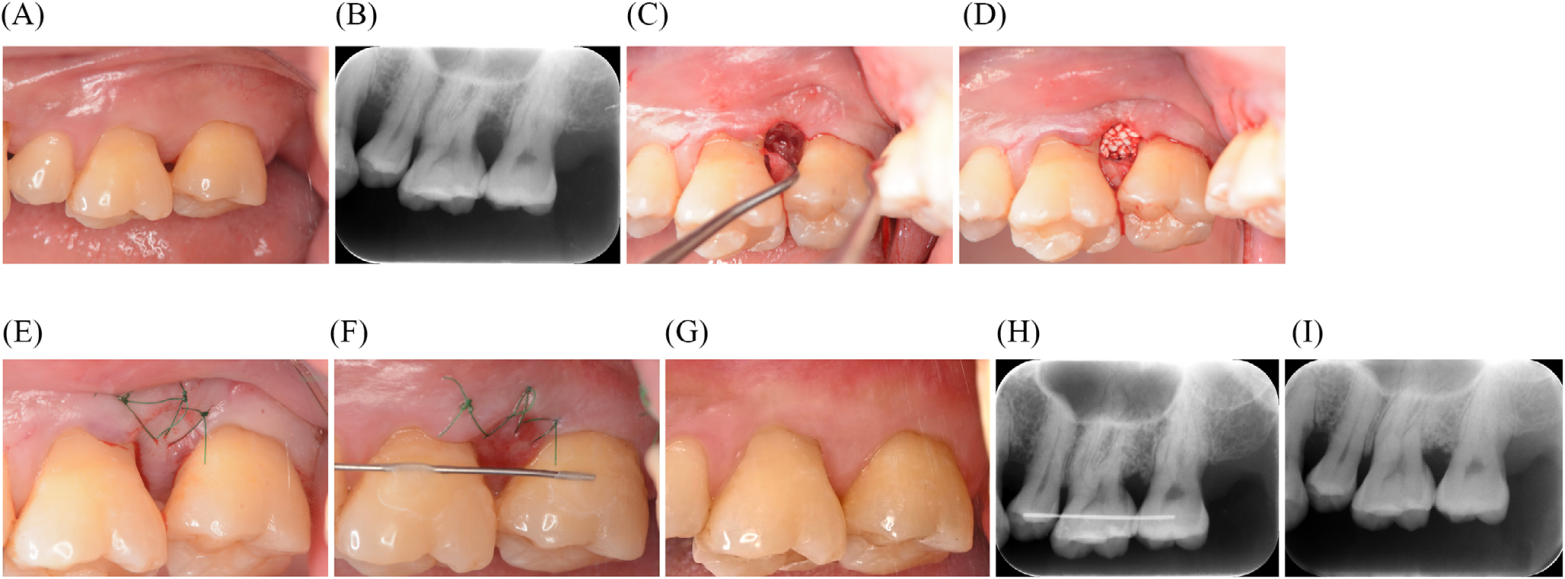
Clinical and radiographic presentation of periodontal regenerative therapy using M‐MIST in the maxillary molar region. (A) Preoperative clinical view of the interdental area between teeth #14 and #15. (B) Preoperative radiograph showing vertical bone defect. (C) Combined bone defect (1‐wall coronal and 2‐wall apical). (D) Placement of carbonate apatite (Cytrans granules). (E) Immediately postoperative buccal view showing primary closure. (F) Buccal view showing primary healing at 1‐week postoperative. (G) Clinical presentation at 1.5‐year follow‐up. (H) Immediately postoperative radiograph. (I) 1.5‐year follow‐up radiograph showing bone fill. M‐MIST, modified minimally invasive surgical technique.

A 47‐year‐old male patient with no history of periodontal treatment presented to Keio University Hospital in April 2023. The patient had no relevant medical or smoking history. After initial periodontal therapy and re‐evaluation, an 8 mm PPD was observed at the buccal aspect in the interdental area between teeth #14 and #15 (Figure 3A). Dental radiograph revealed a vertical bone defect in this area (Figure 3B).

Given that the bone defect was limited to the buccal aspect of the interdental area, periodontal regenerative therapy was performed on teeth #14 and #15 using M‐MIST. The incision line of the interdental papilla was positioned slightly more buccally than conventional MPPT. After making the incision using a microsurgical blade, the interdental papilla was minimally elevated to the extent that the bone defect could be clearly visualized. The bone defect exhibited a combination of a 1‐wall defect at the coronal aspect and a 2‐wall defect at the apical aspect (Figure 3C). The depth of the bone defect was approximately 4 mm. After calculus removal and root surface debridement, Emdogain was delivered, followed by the placement of carbonate apatite granules^§§^ to provide space maintenance (Figure 3D). The flap was repositioned and sutured using reverse cutting; 3/8 Circle 13 mm 6‐0 nylon sutures (Figure 3E,F). Adjacent teeth were splinted immediately postoperatively using wire and resin cement to stabilize the blood clot. The sutures were removed 7 days postoperatively. Good primary healing was achieved. EHI score was 1. At the 1.5‐year postoperative evaluation, PPD improved from 8 to 3 mm (Figure 3G), and dental radiograph showed evidence of bone fill (Figure 3H,I).

In all three cases, patient compliance and plaque control were highly satisfactory. Six months after surgery, periodontal indices indicated substantial clinical improvement, and standardized dental radiographs showed an increase in bone mineral content.

## DISCUSSION

This case study presents two key findings regarding periodontal regenerative therapy of the molar region. First, approaches such as PPT, MPPT, and T‐PAA, in which the incision line is shifted to the buccal or palatal aspect, contribute to enhanced surgical visibility in microsurgical procedures. Second, by considering the extent of papilla elevation, these interdental incision designs can also be applied in the molar region, where SPPF has traditionally been recommended.^10^ These findings are expected to contribute to the success of microsurgical procedures in the molar region, where surgical access and visibility remain major challenges.

In this case study, all therapy was performed in the molar region, where the use of the OM is considered especially necessary. The OM is superior to loupes in terms of magnification, illumination, and ergonomics.^16^, ^17^ In particular, in periodontal regenerative therapy, the advantages of enhanced magnification and coaxial sources of illumination enable clearer and more precise visualization of bone defects and root surfaces. In cases with minimal flap elevation, such as Cases 2 and 3, the OM allows for clear visualization and effective root debridement. Therefore, it may lead to improved primary healing than the use of loupes.

**FIGURE 4:**
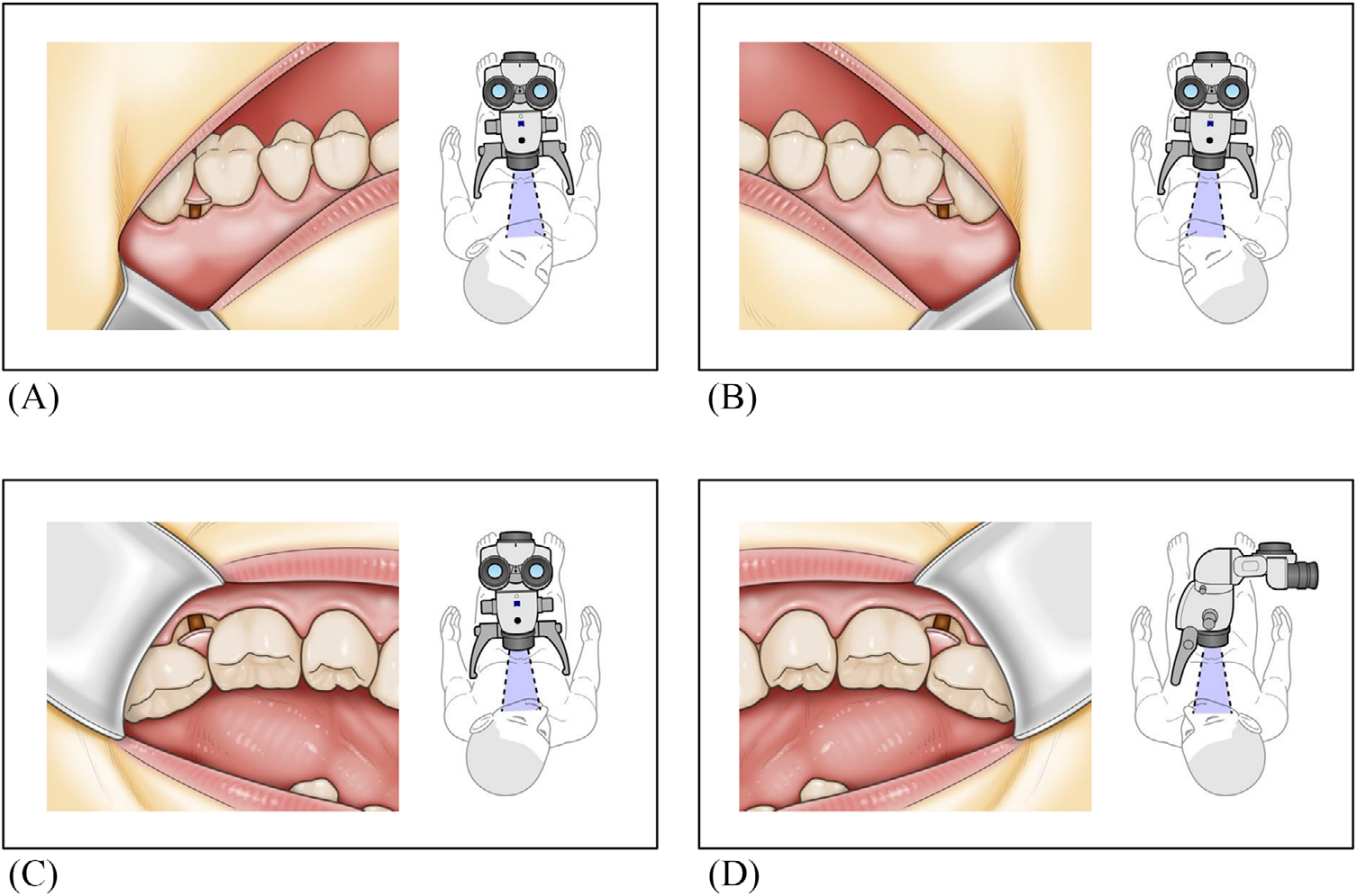
Surgical view in the molar region through operating microscopes. (A) Surgical view and positioning in the maxillary left molar region. (B) Surgical view and positioning in the maxillary right molar region. (C) Surgical view and positioning in the mandibular left molar region. (D) Surgical view and positioning in the maxillary right molar region.

However, compared with that in the anterior and premolar regions, securing visibility and access in the molar region is more challenging. Thus, proper positioning of the OM with regard to the patient and operator must be considered. When properly positioned, the surgical field in the molar region is typically visualized from the buccal direction (Figure 4). Therefore, shifting the interdental incision line to the buccal or palatal aspect is considered highly beneficial for securing adequate surgical visibility. Enhanced visibility may facilitate precise surgical maneuvers and help reduce operation time, which could contribute to reducing postoperative swelling and maintaining the viability of the reflected tissues (Table 1).^18^While elevating the buccal flap can ensure adequate visibility in SPPF, it is likely to disrupt blood supply to the papilla.^19^

**TABLE 1:** Comparison between operating microscopes and loupes.

	Operating microscopes	Loupes
Interdental papilla elevated within the interdental area	Clear visibility	Compromised visibility
The interdental papilla elevated beyond the interdental area	Clear visibility	Sufficient (inferior to OM)
Primary healing	Better than loupes	Not applicable
Ergonomics	Ideal posture	Compromised posture
Learning curve	Challenging	Easier to use

Abbreviation: OM, operating microscopes.

Our findings suggest that interdental incision designs with a buccal or palatal shift, such as PPT, MPPT, and T‐PAA, may represent the preferred approach for microsurgery in the molar region. The next consideration is whether these interdental incision designs can be applied universally to all cases, from the perspective of blood supply.

In the molar region, SPPF has been recommended even when the inter‐root distance exceeds 2 mm because of the greater buccolingual dimension of molars and concerns regarding blood supply.^10^ However, in Cases 2 and 3, favorable primary healing was observed despite shifting the interdental incision to the buccal and palatal aspects. Blood supply to the interdental area is typically derived from three sources: intraosseous, supraperiosteal, and intraligamental arteriole.^20^ In periodontal regenerative therapy, the blood supply from the intraosseous is compromised owing to the presence of bone graft material. The extent of flap elevation likely affects blood supply to the interdental area. In Cases 2 and 3, the interdental papilla was elevated within the interdental area. This suggests that blood supply to the interdental area from the supraperiosteal and intraligamental arteriole on the intact side may have been preserved. However, in Case 1, the flap was extensively elevated beyond the interdental area, which likely affected blood supply from the supraperiosteal and intraligamental arteriole on the intact side (Table 2). Hence, although wound dehiscence was not observed in Case 1, slight gingival recession was noted. These findings suggest that in the molar region, interdental incision designs, such as PPT, MPPT, and T‐PAA, may be applicable in cases where the interdental papilla elevation does not extend beyond the interdental area.

**TABLE 2:** The relationship between blood supply to the interdental area and flap designs.

	Intraosseous arteriole	Intraligamental arteriole	Supraperiosteal arteriole
Extended flap	Affected	Affected	Affected
MIST	Affected	Affected Less effect than extended flap	Affected Less effect than extended flap
M‐MIST	Affected	Preserved (intact area)	Preserved (intact area)

Abbreviations: MIST: minimally invasive surgical technique; M‐MIST: modified minimally invasive surgical technique.

**FIGURE 5:**
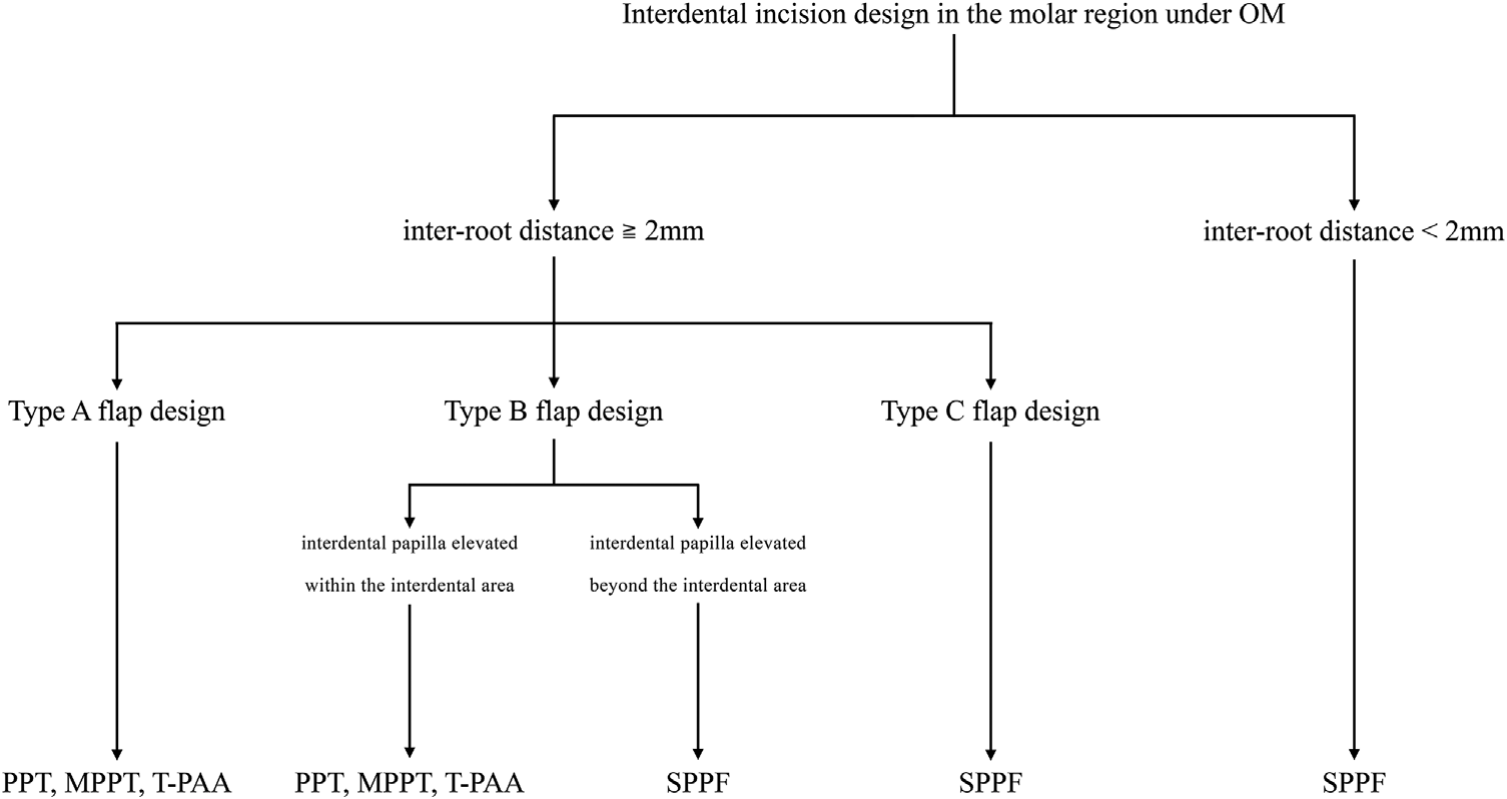
Decision‐making for interdental incision designs in the molar region under operating microscopes. MPPT, modified papilla preservation technique; OM, operating microscope; PPT, papilla preservation technique; T‐PAA, triangle papilla access approach; SPPF, simplified papilla preservation flap.

However, in determining the flap design, not only the blood supply but also the extent of the bone defect is crucial. This is because the flap design must be configured to allow full visualization of the entire bone defect to ensure thorough debridement. In the present three cases, the bone defect morphology was classified as Type III in Case 1, Type II in Case 2, and Type I in Case 3. Accordingly, the flap designs were adapted to each defect type: Type C for Case 1, Type B for Case 2, and Type A for Case 3.^21^ This indicates that flap design is dependent on bone defect morphology. Therefore, accurate preoperative diagnosis of the bone defect morphology, determination of the extent of flap elevation, and selection of the appropriate interdental incision tracing pattern are essential for surgical success (Figure 5). Factors such as oral hygiene, flap tension, and papillary thickness may influence primary closure and warrant consideration during treatment planning. Future studies with larger sample sizes and long‐term follow‐ups would help validate these findings and potentially contribute to the development of guidelines for papillary incision design in different clinical scenarios.

This study has certain limitations. First, the sample size is relatively small, which may limit the generalizability of the findings. Second, quantitative assessment of blood flow was not performed, preventing objective evaluation of vascular dynamics. Future studies with larger cohort sizes and quantitative blood flow analysis using ultrasonographic scans are necessary to validate these findings.

## CONCLUSIONS

This case study suggests that buccal‐ or palatal‐shifted interdental incision designs are effective for molar periodontal regenerative therapy under the OM. Further, papilla incision designs in periodontal regenerative therapy should consider both inter‐root distance and blood supply relative to the extent of papilla elevation. Although SPPF has traditionally been recommended for the molar region, our findings indicate that techniques such as PPT, MPPT, and T‐PAA may also lead to favorable primary healing when the degree of papilla elevation is appropriately considered. Notably, when using the OM, these incision designs are expected to enhance the precision of microsurgical procedures by improving visibility. These observations suggest a potential contribution to improved accuracy and clinical outcomes in microsurgical periodontal regeneration. However, further research with larger sample sizes and long‐term follow‐up is needed to validate these findings and support the development of clinical guidelines for papillary incision design across a variety of treatment scenarios.

## AUTHOR CONTRIBUTIONS

Shunichi Shibazaki performed periodontal regeneration. Shunichi Shibazaki and Satoru Morikawa contributed to the data analysis, manuscript preparation, and final manuscript approval. All authors have read the final version of the manuscript and provided their approval.

## CONFLICT OF INTEREST STATEMENT

The authors declare no conflicts of interest.

## Supporting information



Debridement of the 2‐wall bone defect. Surgical view showing the debridement process of the 2‐wall intrabony defect at the mesial aspect of tooth #2.

Final appearance of the debrided defect. View of the cleaned root surface and the 2‐wall defect configuration after completion of debridement.

Application of regenerative materials. Application of enamel matrix derivative (Emdogain) followed by the placement of deproteinized bovine bone mineral (DBBM) into the defect.

Wound closure. Repositioning of the palatal flap and suturing with 6‐0 nylon sutures to achieve primary wound closure.

## Data Availability

The data that support the findings of this study are available from the corresponding author upon reasonable request.
